# Genetic variation in recombination rate in the pig

**DOI:** 10.1186/s12711-021-00643-0

**Published:** 2021-06-25

**Authors:** Martin Johnsson, Andrew Whalen, Roger Ros-Freixedes, Gregor Gorjanc, Ching-Yi Chen, William O. Herring, Dirk-Jan de Koning, John M. Hickey

**Affiliations:** 1 The Roslin Institute and Royal (Dick) School of Veterinary Studies, The University of Edinburgh, Midlothian, EH25 9RG Scotland, UK; 2 Department of Animal Breeding and Genetics, Swedish University of Agricultural Sciences, P.O. Box 7023, 750 07 Uppsala, Sweden; 3 Departament de Ciència Animal, Universitat de Lleida-Agrotecnio-CERCA Center, Lleida, Spain; 4 Pig Improvement Company, Genus plc, 100 Bluegrass Commons Blvd., Ste2200, Hendersonville, TN 37075 USA

## Abstract

**Background:**

Meiotic recombination results in the exchange of genetic material between homologous chromosomes. Recombination rate varies between different parts of the genome, between individuals, and is influenced by genetics. In this paper, we assessed the genetic variation in recombination rate along the genome and between individuals in the pig using multilocus iterative peeling on 150,000 individuals across nine genotyped pedigrees. We used these data to estimate the heritability of recombination and perform a genome-wide association study of recombination in the pig.

**Results:**

Our results confirmed known features of the recombination landscape of the pig genome, including differences in genetic length of chromosomes and marked sex differences. The recombination landscape was repeatable between lines, but at the same time, there were differences in average autosome-wide recombination rate between lines. The heritability of autosome-wide recombination rate was low but not zero (on average 0.07 for females and 0.05 for males). We found six genomic regions that are associated with recombination rate, among which five harbour known candidate genes involved in recombination: *RNF212*, *SHOC1*, *SYCP2*, *MSH4* and *HFM1*.

**Conclusions:**

Our results on the variation in recombination rate in the pig genome agree with those reported for other vertebrates, with a low but nonzero heritability, and the identification of a major quantitative trait locus for recombination rate that is homologous to that detected in several other species. This work also highlights the utility of using large-scale livestock data to understand biological processes.

**Supplementary Information:**

The online version contains supplementary material available at 10.1186/s12711-021-00643-0.

## Background

Meiotic recombination results in the exchange of genetic material between homologous chromosomes. After chromosomes have paired up and duplicated, they can break and exchange segments of chromosomes. Such recombination events are not evenly distributed along the chromosomes and result in a variable landscape of recombination rate across the genome, with peaks and troughs.

The landscapes of recombination rate among vertebrate genomes share several features. Recombination rate tends to be lower in the middle of chromosomes and higher near their ends (reviewed by Stapley et al. [1]). Recombination rate is positively correlated with the fraction of guanine and cytosine bases (GC content), which is likely due to GC-biased gene conversion that favours alleles with a higher GC content and is promoted by recombination [2, 3]. Recombination rate has also been found to be associated with the presence of repeat elements, with different repeat elements being biased towards or away from high-recombination regions [4–6]. Recombination rate also differs between sexes and is typically higher in females than in males, except at chromosome ends [3, 7–9]. At a finer scale, most recombination events occur in regions of only a few kb that are called recombination hotspots [6, 10]. To a large extent, the location of recombination hotspots is driven by PRDM9 (a zinc finger protein with histone methyltransferase activity that directs meiotic recombination to specific DNA-binding sites by its zinc finger array). There is direct evidence of PRDM9-driven hotspot targeting in humans and mice [11–14], but evolutionary comparisons suggest that the process is shared widely across vertebrates [15].

Previous analyses [16–18] of the recombination landscape of the pig genome have revealed that it shares broadly the following features with other vertebrate genomes: a low recombination rate in the middle of chromosomes, a correlation between recombination rate and GC content, and a difference in recombination rate between males and females. However, in the pig genome, the sex difference in recombination rate is unusual in that the recombination rate is mostly higher in females even near the ends of chromosomes. The pig karyotype has both acrocentric chromosomes, with the centromere at one end, and non-acrocentric chromosomes. On the pig’s acrocentric chromosomes, recombination rate is higher near both ends, although the centromere is located at one end, which has been confirmed by direct counting of recombination events using immunohistochemistry [19]. Like most mammals, the pig has a full-length *PRDM9* gene that displays rapid evolution of its DNA-binding zinc finger array, which suggests that the pig genome contains *PRDM9*-dependent recombination hotspots [15]. In this paper, we investigated how recombination rate on a broad scale varies between individuals and populations in the pig. Recombination rates have been shown to be highly variable in several species, with studies in humans [20, 21], mice [22, 23], cattle [24–27], deer [28, 29], sheep [30, 31] and chickens [32] showing that recombination rate is genetically variable, and identifying genetic associations with alleles at a handful of genes that are involved in meiosis, including *RNF212*, *MSH4*, *REC8* and *PRDM9* (reviewed by [1, 33]).

Analysis of the genetic basis of recombination rate requires estimates of recombination rate from a large number of related individuals. Recombination rate can be estimated by phasing genotypes in pedigrees [8, 34–36], by direct counting in gametes [19, 37], or by measuring linkage disequilibrium in population samples [10]. Counting-based methods require specific experiments. Linkage disequilibrium-based methods only provide average values of recombination rate for a population but have the benefit that they can estimate fine-scale recombination landscapes, including recombination hotspots, while pedigree-based methods can only estimate broad-scale recombination rate, averaged over much longer distances. In this paper, we used a new pedigree method based on multilocus iterative peeling [38, 39] to estimate recombination rates simultaneously with genotype imputation. This allowed us to use data from a pig breeding programme, for which animals were genotyped with marker panels of varying density for genomic selection.

In this work, we assessed genetic variation in recombination rate along the genome and between individuals in the pig using multilocus iterative peeling on 150,000 individuals across nine genotyped pedigrees. We used these data to estimate the heritability of recombination and perform a genome-wide association study of recombination in the pig.

## Methods

We analysed the landscape of recombination rate in the genome of nine lines of pigs from a commercial breeding programme. We performed six analyses: (1) an analysis of the average number of recombination events on each chromosome (the genetic length of chromosomes) to estimate between-sex and between-line differences in genetic length and then compared these estimates to previously published estimates; (2) an analysis of the distribution of recombination events along the chromosomes (landscapes of recombination rates) to estimate between-line and between-sex differences; (3) estimation of the correlation between recombination rate and DNA sequence features that are known to correlate with recombination rate; (4) estimation of pedigree-based and genomic heritabilities of recombination rate; (5) a genome-wide association study to detect chromosomal regions associated with recombination rate; and (6) a simulation to test the accuracy of the inference method.

### Data

The data consisted of single nucleotide polymorphism (SNP) chip genotype and pedigree data from nine commercial pig breeding populations of varying sizes with overlapping generations from the Pig Improvement Company breeding programme, covering 20–30 generations. These lines represent broadly-used populations, including animals of Large White, Landrace, Duroc, Hampshire and Pietrain heritage. Table 1 shows the number of individuals used for recombination inference and the number of parents used for heritability estimation and genome-wide association analyses for each line. The pigs were either genotyped at low density (15 K SNPs) using the GGP-Porcine LD BeadChip (GeneSeek, Lincoln, NE) or at high density (50 K, 60 K, 80 K SNPs) using the GGP-Porcine HD BeadChips (GeneSeek, Lincoln, NE). In total, genotype data were available for 390,758 pigs, among which 39% were genotyped on the higher density chips, 51% on the low-density chip, and 10% were ungenotyped.

**Table 1:** Description of the data

Line	Pedigree size	Number of individuals with estimates of recombination rate	Dams	Sires
1	69 k	23,273	2651	347
2	64 k	16,661	2255	368
3	34 k	14,278	2169	215
4	18 k	7153	1239	163
5	70 k	33,566	4349	293
6	34 k	11,666	1971	162
7	15 k	263	76	20
8	22 k	4177	727	78
9	108 k	34,726	5171	492

The table shows the size of the pedigrees, number of individuals that passed filtering and for which recombination estimates were included, and the number of dams and sires of these individuals used for heritability estimation and genome-wide association. By necessity, we inferred recombination rates from an equal number of maternal and paternal chromosomes, but they derive from a much larger number of dams than sires

### Estimation of recombination rate using multilocus iterative peeling

Multilocus iterative peeling was used to estimate the number and location of recombination events in each individual [38–40]. Multilocus iterative peeling uses pedigree and genotype data to infer the phased genotype of each individual by calculating the probability of each genotype state based on the individual’s own genetic data, the genotypes of their parents (“anterior” probabilities), and the genotypes of their offspring (“posterior” probabilities) [41]. Multilocus iterative peeling tracks which parental haplotype an individual inherits at each locus (referred to as segregation probabilities) and uses this information to determine which parental allele an individual inherits, particularly from parents that are heterozygous for that allele. Segregation probabilities can be used to determine the number and location of likely recombination events. When a recombination occurs, the haplotype that an individual inherits from one parent will change, which causes the inferred segregation probabilities to change. By analysing the joint distribution of the segregation probabilities at two neighbouring loci, the expected number of recombination events between two loci and that across an entire chromosome can be estimated. In our study, we introduced two simplifications to the multilocus peeling method of [40], in order to estimate recombination rates and reduce runtime and memory requirements: (1) we calculated the segregation probabilities and the “anterior” probabilities separately for each parent instead of modelling their full joint distribution; and (2) we called the segregation and genotype probabilities of the offspring when estimating the “posterior” probability for each parent, taking them as certain where they were above thresholds of 0.99 and 0.90 for the segregation and genotype probabilities, respectively. Segregation and genotype probabilities that did not reach the threshold were set to missing, implying that inheritance of either parental haplotype and all genotype states, respectively, are equally likely. By calling the segregation and genotype values, we were able to store many of the calculations in lookup tables instead of re-computing them for each locus and each individual. In addition, calling the segregation values reduced the chance that feedback loops occurred between offspring with fractional segregation values at multiple nearby loci.

The joint distribution of segregation values depends on chromosome length (in cM). To estimate chromosome length, we started with a length of 100 cM (on average 1 recombination per chromosome), and then refined this estimate in a series of steps. At each step, we calculated the expected number of recombination events for each individual at each locus, and set the chromosome length based on the average population recombination rate. This step was repeated four times. In preliminary simulations, we found that the estimates of chromosome length converged after four iterations and that the estimates of recombination rate for target individuals were insensitive to the assumed chromosome length.

### Filtering of individuals

After estimation of recombination rates, we filtered the data by removing individuals without genotyped parents and grandparents in order to focus on individuals with high-quality estimates of recombination rate. An additional seven individuals with extremely high average recombination rate estimates (> 5 cM/Mbp) were also removed. These filtering steps reduced the number of pigs to 145,763. Table 1 shows the resulting number of individuals with estimates of recombination rate per line, and among these, the number of dams and sires used for heritability estimation and genome-wide association analyses.

### Comparison of recombination landscapes between lines and with the literature

To compare the recombination landscapes of the nine lines, we calculated between-line pairwise correlations of the estimated recombination rates at each marker interval, within each sex. To compare the recombination landscapes between females and males, we calculated the correlation of recombination rates between each pair of SNPs between sexes within each line. We compared genetic map lengths between lines using a linear model by fitting the number of recombination events observed on a chromosome as response variable and fixed effects for each line and chromosome. Lines were compared separately for each sex. To compare the recombination landscapes obtained in our study to results in the literature, we plotted the genetic map length for each chromosome against published genetic map lengths [16].

### Correlations with genomic features

To investigate the relationship of local recombination rates with genomic features, we divided the autosomal part of the Sscrofa11.1 genome [42] into 2272 windows of 1 Mb. We used the software Biostrings version 2.52.0 (https://bioconductor.org/packages/Biostrings) and TFBSTools version 1.22.0 [43] in the R statistical environment to estimate four features of sequence composition for each 1-Mb window: (1) the fraction of guanine and cytosine bases (GC content); (2) the count of the *PDRM9* consensus motif CCNCCNTNNCCNC [44]; (3) the count of the predicted porcine *PRDM9* motif; and (4) the count of the CCCCACCCC motif, which was the most strongly associated motif with recombination rate in the pig reported by[16].

In order to predict the porcine PRDM9 motif, we used the online Cys_2_His_2_ Zinc Finger predictor of [45] and the amino acid sequence (accession number XP_013849667) identified by [15] as pig PRDM9, although it was annotated by the NCBI gene annotation (release 105) as PRDM7. Applying the polynomial SVM predictor to nine clustered zinc finger domains toward the end of the sequence results in a 25-bp motif that partially matches the consensus PRDM9 motif [see Additional file 1 Figure S1]. To detect such matches, we used the TFBSTools software, with a minimum score of 70% of the maximum score.

We used repeat data from RepeatMasker (http://www.repeatmasker.org) [46] from the pig genome to estimate the density of repeats in the same 1-Mb windows and subdivided the total content of repeats into five broad categories: (1) long interspersed elements (LINE); (2) fraction of short interspersed elements (SINE); (3) long terminal repeats (LTR); (4) DNA repeats elements; and (5) low complexity repeats. Then, we calculated the correlation of the recombination rate of each window with each sequence feature.

To find putative pericentromeric regions, we used the inferred centromere positions from [42]. For chromosomes 8, 11 and 15, for which more than one location that were far apart from each other was inferred, we picked the most likely location based on the pig karyotypes reported in [47].

### Heritability of autosome-wide recombination rate

We estimated the narrow-sense heritability of the autosome-wide recombination rate per Mb of parents that had genotyped offspring using the animal model in the MCMCglmm package [48] version 2.29. The animal model included an additive genetic effect for the parent based on pedigree relatedness and a permanent environmental effect for each parent as random effects, and no additional fixed effect covariates. Because we measured recombination rate in parents with varying numbers of genotyped offspring (see Table 1), we used a model with repeated observations and a permanent environmental effect for each parent. We analysed each sex and line separately. We used parameter expanded priors [49] for the variance of permanent environmental effects and for the additive genetic effects, using V = 1, ν = 1, αμ = 0, αV = 1000, which corresponds to a half-Cauchy prior with a scale of 100, and an inverse-Wishart prior (V = 1, ν = 1) for the residual variance. Line 7 was excluded from the heritability estimation because of its small number of dams and sires.

### Genome-wide association

We performed genome-wide association studies of autosome-wide recombination rates using a hierarchical linear mixed model in the RepeatABEL package [50] version 1.1. The linear mixed model used a genomic relationship matrix to account for relatedness and included a random permanent environmental effect for each parent, and no further fixed effects beyond the SNP being considered. That is, the genome-wide association analysis was performed with the same model as above, except that it used a genomic relationship matrix and fitted each SNP separately as fixed effect. The test statistic was estimated simultaneously for all SNPs by an approximation using eigendecomposition [50]. We analysed each sex and line separately. The genotype data were imputed to best-guess genotypes from the same run of multilocus peeling that was used for estimating the number of recombination events. Line 7 was again excluded from this analysis. We report SNPs below a conventional threshold of $p<5 \times {10}^{-8}$ (commonly used in large-scale genome-wide association studies in humans and livestock, and likely to be conservative [51, 52]) as significant. When there were more than one significant SNP within a megabasepair (Mb) region, we used the most significant SNPs to report the explained variance and the frequency of the allele that is associated with the higher recombination rate. In the case of ties of the most significant SNPs, we selected the SNP that was closest to the mean position of the most significant SNPs. We report the gene that was closest to the most significant SNP based on the Ensembl Genes database version 102, as well as any candidate genes that are known to be involved in recombination, based on [53]. To do this, we searched for the location of the pig homologs of recombination-associated genes analysed in [53] and report those that are located within a few Mb of the significant SNPs.

### Meta-analysis of genome-wide association studies

We performed a meta-analysis of the genome-wide association studies by combining the lines but analysing sexes separately, using the meta R package 4.17-0 [54]. It is based on an inverse variance weighting and a fixed-effects meta-analysis that takes the estimated marker effects and standard errors from RepeatABEL as input. We report significant SNPs that have a p-value lower than a conventional threshold of 5 × 10^−8^.

### Simulations

To demonstrate that the method for estimating recombination rate by multilocus peeling works, we tested it first on a simulated dataset with features similar to the real data. We simulated genotype data using the AlphaSimR 0.10.0 software [55] for one chromosome, using the same pedigree and the same number of genotyped SNPs (1522) as for the largest of the nine lines. We used the MaCS coalescent simulator [56], as included in AlphaSimR, to generate founder haplotypes. We used the “GENERIC” population history of AlphaSimR, where MaCS generates founder haplotypes from a population history with decreasing effective population size over time, reflecting the history of domestication and selective breeding of livestock species. Then, we created a variable recombination landscape by modifying the genetic distances between the resulting markers. The modified recombination landscape had a constant recombination rate in the middle of the chromosome (between 30 and 70% of the original map), and two regions of high recombination rate at the chromosome ends (the first and last 30%, respectively), described by second degree polynomials. Sex-specific recombination rates were set to be 1.3 times higher in females than in males. We assessed the accuracy of the inferred recombination landscape by calculating the correlation between the estimated number of recombination events at each marker interval and the true number of recombination events. We also calculated the correlation between the estimated number of recombination events and a smoothed recombination landscape, where values were averaged in non-overlapping 50 SNP windows.

## Results

Our results show that: (1) the genetic length of chromosomes differs between sexes and lines; (2) the recombination landscape is similar between lines but differs between sexes; (3) as previously reported, the local recombination rate is correlated with GC content, repeat content, the CCCCACCCC sequence motif, but we do not confirm the previously described correlation with the *PRDM9* consensus motif; (4) the heritability of recombination rate was on average 0.07 for females and 0.05 for males; and (5) six regions of the genome were associated with recombination rate, of which five contained known candidate genes, i.e. *RNF212*, *SHOC1*, *SYCP2*, *MSH4* and *HFM1*.

In the simulation analysis, we found that multilocus iterative peeling could estimate the number of recombination events per individual with an accuracy of 0.7 for dams and 0.5 for sires, as well as the average recombination landscape along a chromosome, but with a tendency to overestimate the genetic length.

### Differences in genetic map length between lines and sexes

**Fig. 1:**
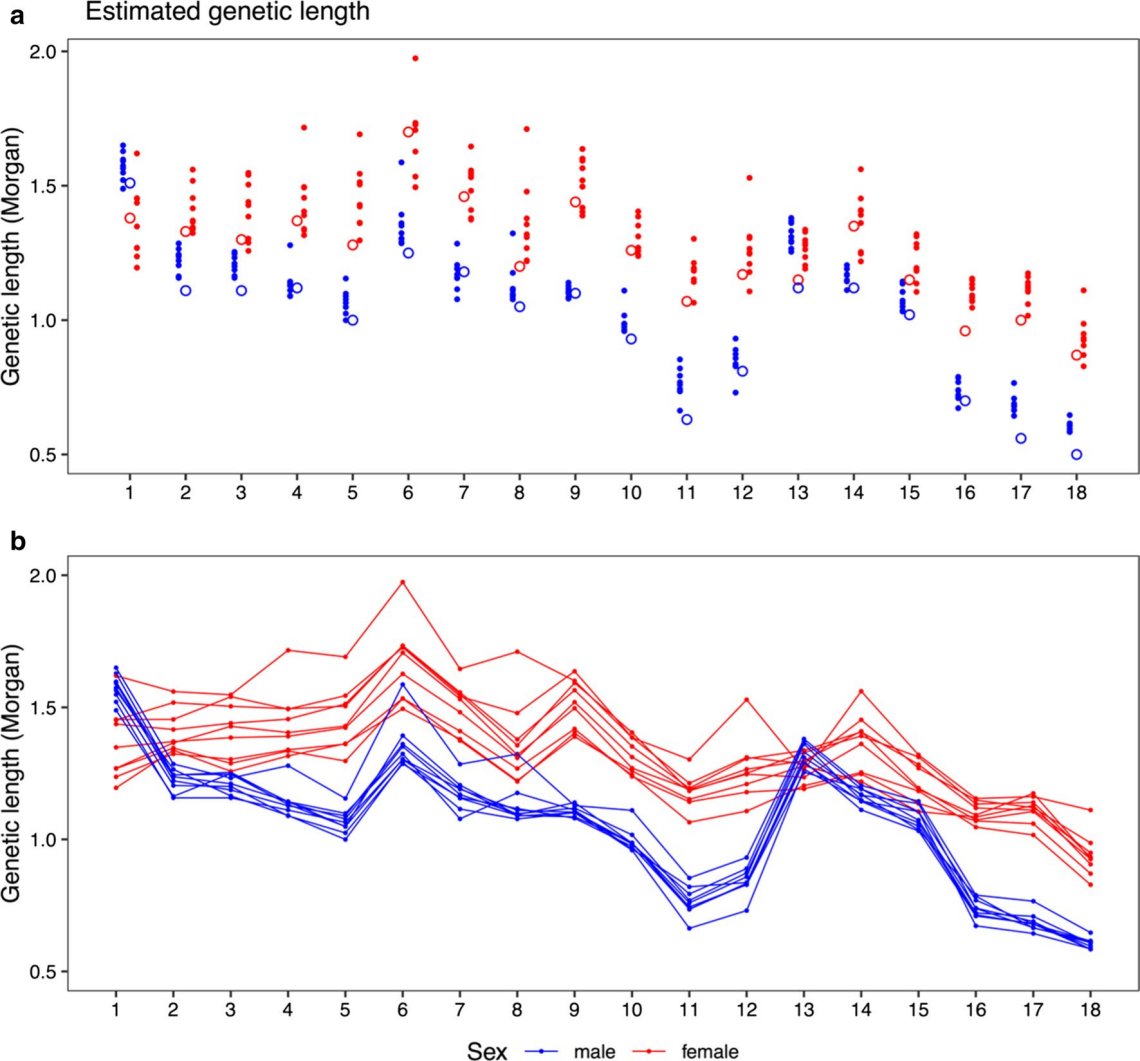
Genetic length of pig autosomes estimated by multilocus iterative peeling. The horizontal axis corresponds to chromosomes 1–18. Red dots and lines are estimates for females and blue dots and lines are estimates for males. **a** compares estimates from multilocus iterative peeling (filled dots) to estimates from [1] (open circles). **b** shows the same data, using lines to connect estimates from the same line of pigs

The genetic length of chromosomes differed between lines and sexes. Figure 1 shows the estimated map length of each chromosome compared with previously published estimates [16]. Table 2 provides the estimated total map length for each sex and line, with confidence intervals derived from the linear model. On average, the estimated sex-averaged map was 21.5 Morgan (M) (0.95 cM/Mb) and the estimated female and male maps were 23.6 M (1.04 cM/Mb) and 19.5 M (0.86 cM/Mb), respectively. Tables S1–S3 [see Additional file 2: Tables S1, Additional file 3: Table S2 and Additional file 4: Table S3] contain male, female, and sex-averaged maps of the pig recombination landscape, respectively.

**Table 2:** Estimates of total map length

Line	Sex	Map length (Morgan)	Lower	Upper	Rate (cM/Mbp)
1	Female	23.6	23.5	23.6	1.04
1	Male	19.4	19.4	19.5	0.86
2	Female	24.1	24.1	24.2	1.06
2	Male	20.0	20.0	20.0	0.88
3	Female	22.3	22.2	22.3	0.98
3	Male	18.2	18.1	18.2	0.80
4	Female	23.5	23.4	23.5	1.04
4	Male	19.3	19.3	19.4	0.85
5	Female	22.8	22.7	22.8	1.01
5	Male	18.7	18.6	18.7	0.82
6	Female	23.7	23.6	23.7	1.04
6	Male	19.5	19.5	19.6	0.86
7	Female	25.9	25.5	26.2	1.14
7	Male	21.7	21.4	22.1	0.96
8	Female	24.1	24.0	24.2	1.06
8	Male	20.0	19.9	20.1	0.88
9	Female	22.6	22.6	22.6	1.00
9	Male	18.5	18.4	18.5	0.82
Average	Female	23.6			1.04
	Male	19.5			0.86
	Sex-average	21.5			0.95

Intervals are 95% confidence intervals

Our estimates of the genetic lengths of chromosomes were comparable to previously reported estimates, but tended to be longer. We found that females had a higher recombination rate, except on chromosome 1, for which the male recombination rate was higher, and on chromosome 13, for which the recombination rate was similar for both sexes. This confirms previous results [16].

### Differences in the recombination landscape between sexes

**Fig. 2:**
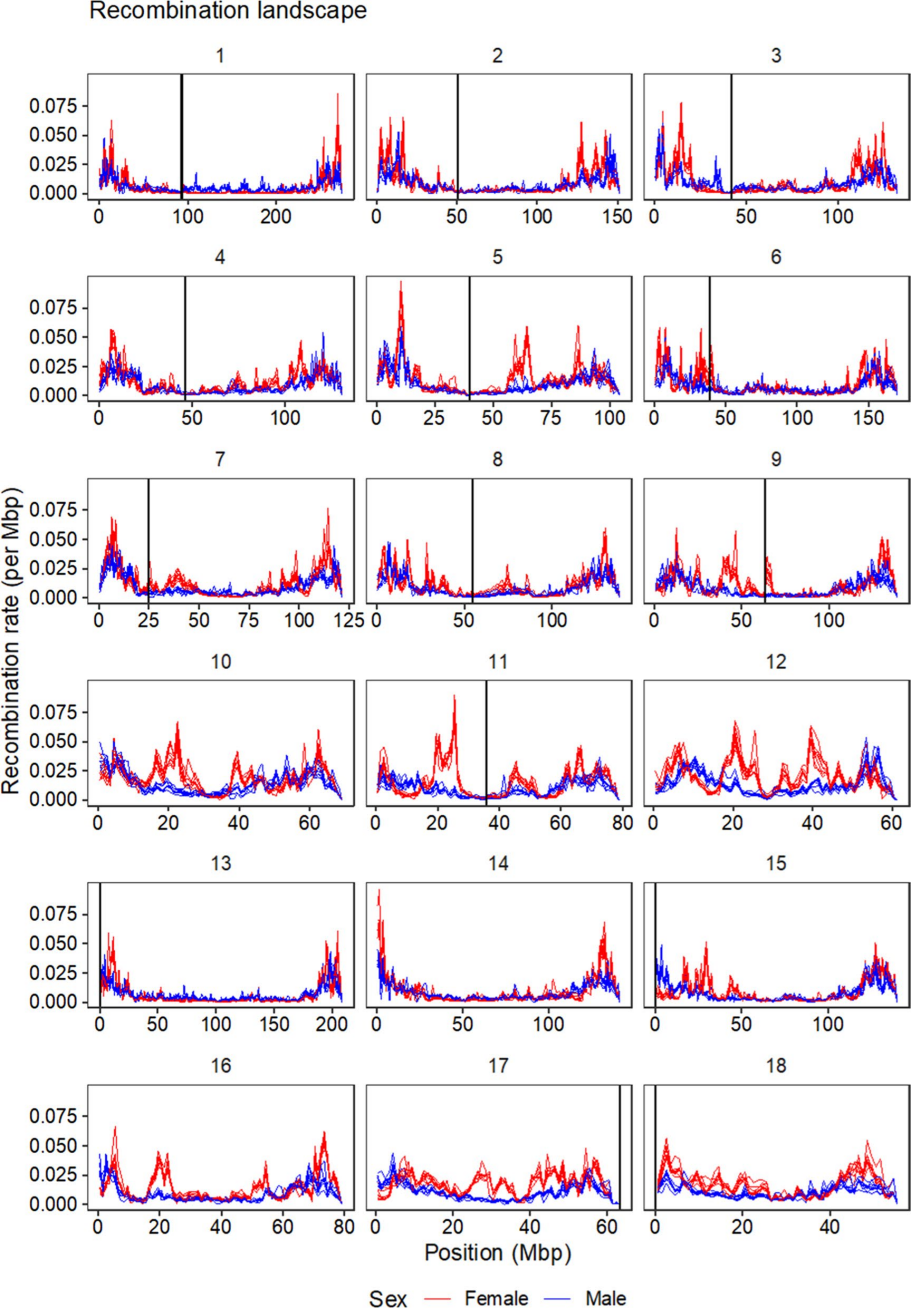
Recombination landscape of the pig genome. The lines show recombination rate in 1-Mb windows along the pig genome (Sscrofa11.1). Red lines are estimates for females and blue lines are estimates for males. Each line corresponds to one of the nine breeding lines. The black vertical lines indicate predicted centromere locations in the reference genome, for chromosomes for which the information is available

**Fig. 3:**
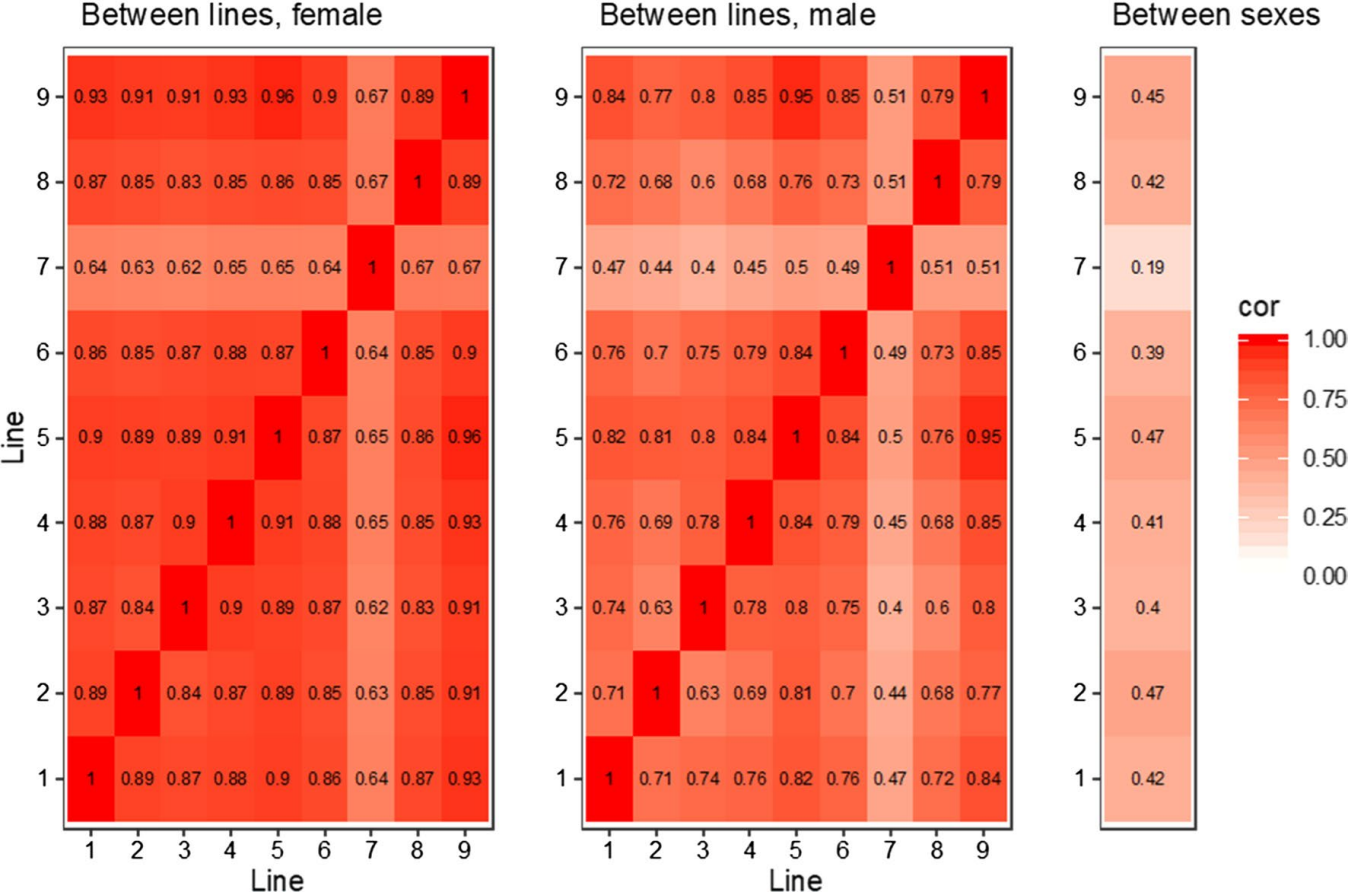
Correlation heatmap of recombination landscapes between lines and sexes. Heatmaps show pairwise correlations between lines of the estimated recombination rates at each marker interval, within each sex, and the correlation between sexes within each line

The pattern of the recombination landscape was similar between lines but differed between the sexes. Figure 2 presents the landscape of the recombination rate for each chromosome, whereas Fig. 3 shows the pairwise correlations of the recombination rate estimates at each marker interval between lines for each sex, as well as the pairwise correlations between sexes within each line. Both sexes had higher recombination rates near the ends of chromosomes and lower recombination rates in the middle of the chromosomes. However, there were several broad regions that had a high recombination rate in females but not in males and these regions were repeatable between lines. The mean between-line correlation was 0.83 in females and 0.70 in males, while the mean correlation between sexes was 0.40 across lines.

### Correlations of recombination rates with genomic features

**Fig. 4:**
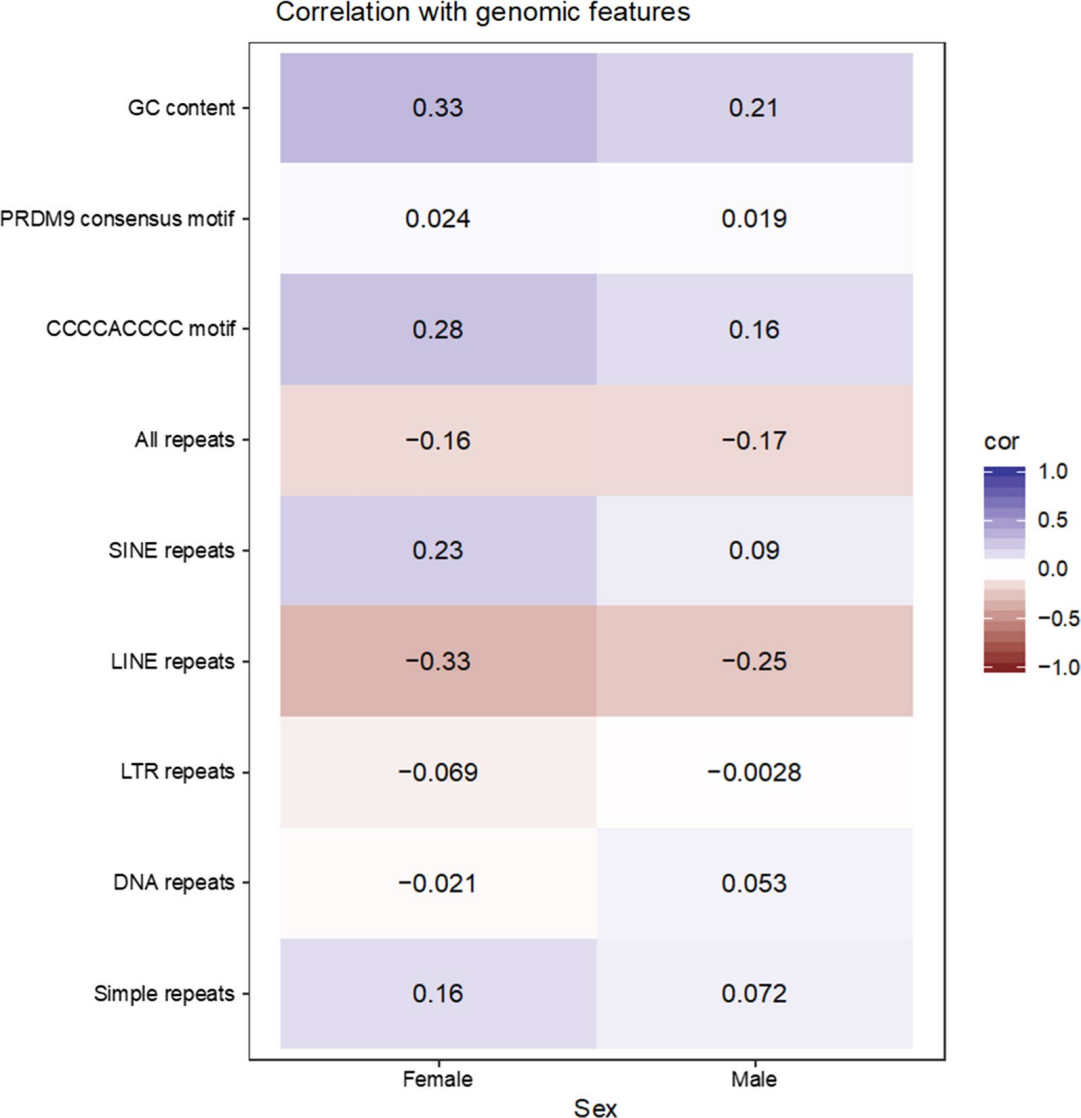
Heatmap of correlations of recombination rates with genomic features in windows of 1 Mb. The heatmap shows correlations of recombination rates with sequence features within 2272 1-Mb windows along the autosomes of the pig genome (Sscrofa11.1)

Figure 4 shows the correlations between recombination rate and genomic features in 1-Mb windows, for each sex. The correlation of local recombination rates with GC content, sequence repeats, and particular sequence motifs was moderate to low (absolute values less than 0.33). When all classes of repeats were combined, correlations were positive with GC content and negative with sequence repeats. The correlation between recombination rate and different types of repeats was variable. Recombination rate was only weakly correlated with counts of the *PRDM9* consensus motif CCNCCNTNNCCNC (0.024 in females and 0.019 in males), negatively correlated with counts of the predicted porcine *PRDM9* motif (− 0.22 in females and − 0.17 in males), but moderately positively correlated with counts of the CCCCACCCC motif (0.28 in females and 0.16 in males), which was previously reported to be enriched in high recombination regions in the pig genome [16].

### Heritability of recombination rate

**Fig. 5:**
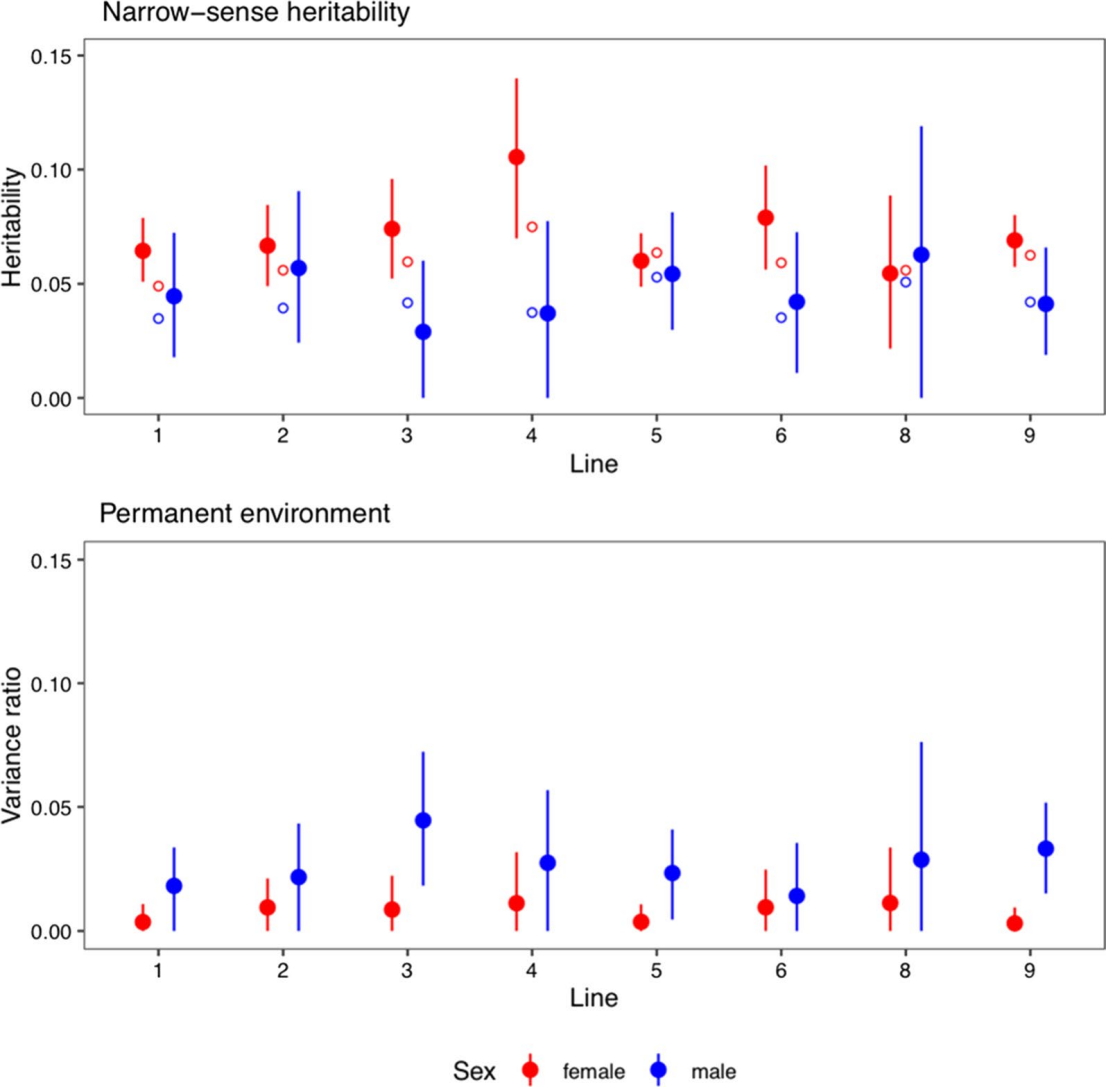
Heritability of genome-wide recombination rates. The dots are estimates of narrow-sense heritability and of the permanent environmental effect variance proportion for genome-wide recombination rates based on an animal model, with 95% credible intervals. Red and blue are female and male estimates, respectively. Open circles show estimates of genomic heritability based on the genome-wide association analyses. Line 7 was excluded from the analysis because of its small number of dams and sires

Figure 5 shows estimates of heritability and of the proportion of permanent environmental variance by sex and line. The autosome-wide recombination rate had a low but non-zero heritability, on average 0.07 for females and 0.05 for males, with the lower limit of the confidence interval close to zero only for male estimates in three lines. The open circles in Fig. 5 show estimates of genomic heritability from the genome-wide association analyses. The genomic heritabilities suggest that the SNP chip captured most (on average 83%) of the additive genetic variance of recombination rate.

### Genome-wide association analysis of recombination rate

**Fig. 6:**
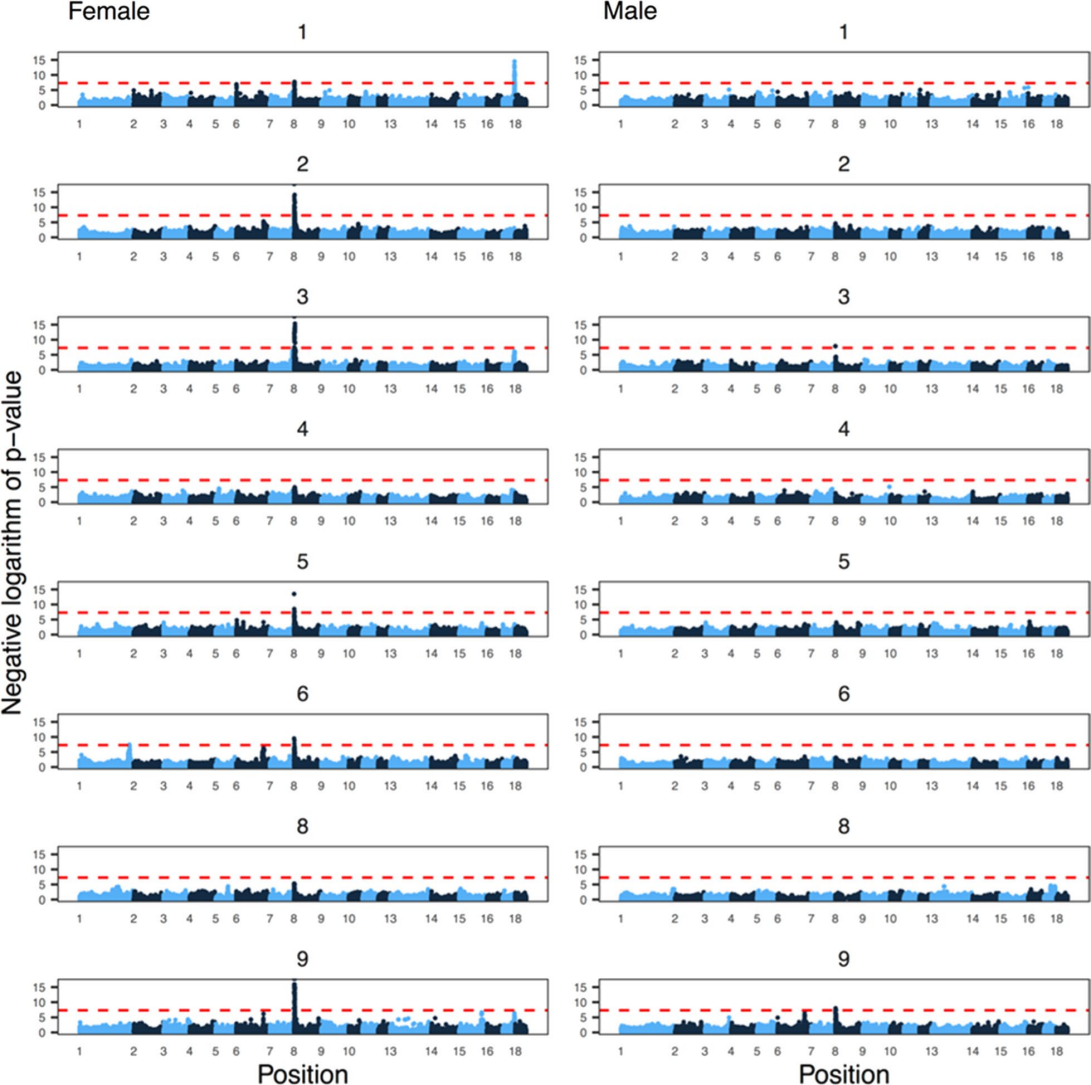
Genome-wide association analysis of the genome-wide recombination rate. The subplots are Manhattan plots of the negative logarithm of the p-value of association against genomic position, broken down by line and sex. Alternating colours correspond to chromosomes 1 to 18. Line 7 was excluded from the analysis because of its small number of dams and sires. The dashed red line shows a conventional genome-wide significance threshold of 5 × 10^−8^. The numbers for chromosomes 11, 12 and 17 were removed for legibility

Genome-wide association studies, performed separately for each line, revealed three regions of the pig genome that contained SNPs that were associated with the autosome-wide recombination rate. Figure 6 shows the genome-wide association results within each line, broken down by sex. Table 3 shows the location of the most significant SNP for each region with the amount of variance explained, its allele frequency, and the closest gene based on the Ensembl database. We identified one region that was associated with female recombination rate at the beginning of chromosome 8 in six of the lines, one region on chromosome 17 in line 1, and one on chromosome 1 in line 6. The region on chromosome 8 was also associated with male recombination rate in two of the lines.

**Table 3:** Genome-wide association study hits for genome-wide recombination rate, with position of the most significant SNP, additive genetic variance explained by the lead (most significant) SNP, and frequency of the allele associated with the higher recombination rate

Chr	Sex	Line	Lead SNP position	Gene closest to lead SNP	Genetic variance explained	Allele frequency
1	Female	6	252,547,401	SHOC1 (ENSSSCG00000005463)	0.10	0.57
8	Female	1	2,253,270	lncRNA ENSSSCG00000047394	0.08	0.90
8	Female	2	75,256	PDE6B (ENSSSCG00000036645)	0.60	0.53
8	Female	3	226,298	CPLX1 (ENSSSCG00000037527)	0.41	0.70
8	Male	3	226,298	CPLX1 (ENSSSCG00000037527)	0.44	0.74
8	Female	5	259,617	GAK1 (ENSSSCG00000021289)	0.07	0.27
8	Female	6	259,617	GAK1 (ENSSSCG00000021289)	0.12	0.74
8	Female	9	75,256	PDE6B (ENSSSCG00000036645)	0.14	0.12
8	Male	9	1,283,621	ZFYVE28 (ENSSSCG00000008689)	0.22	0.41
17	Female	1	59,968,884	FAM217B (ENSSSCG00000007531)	0.16	0.78

*Chr* chromosome number

**Fig. 7:**
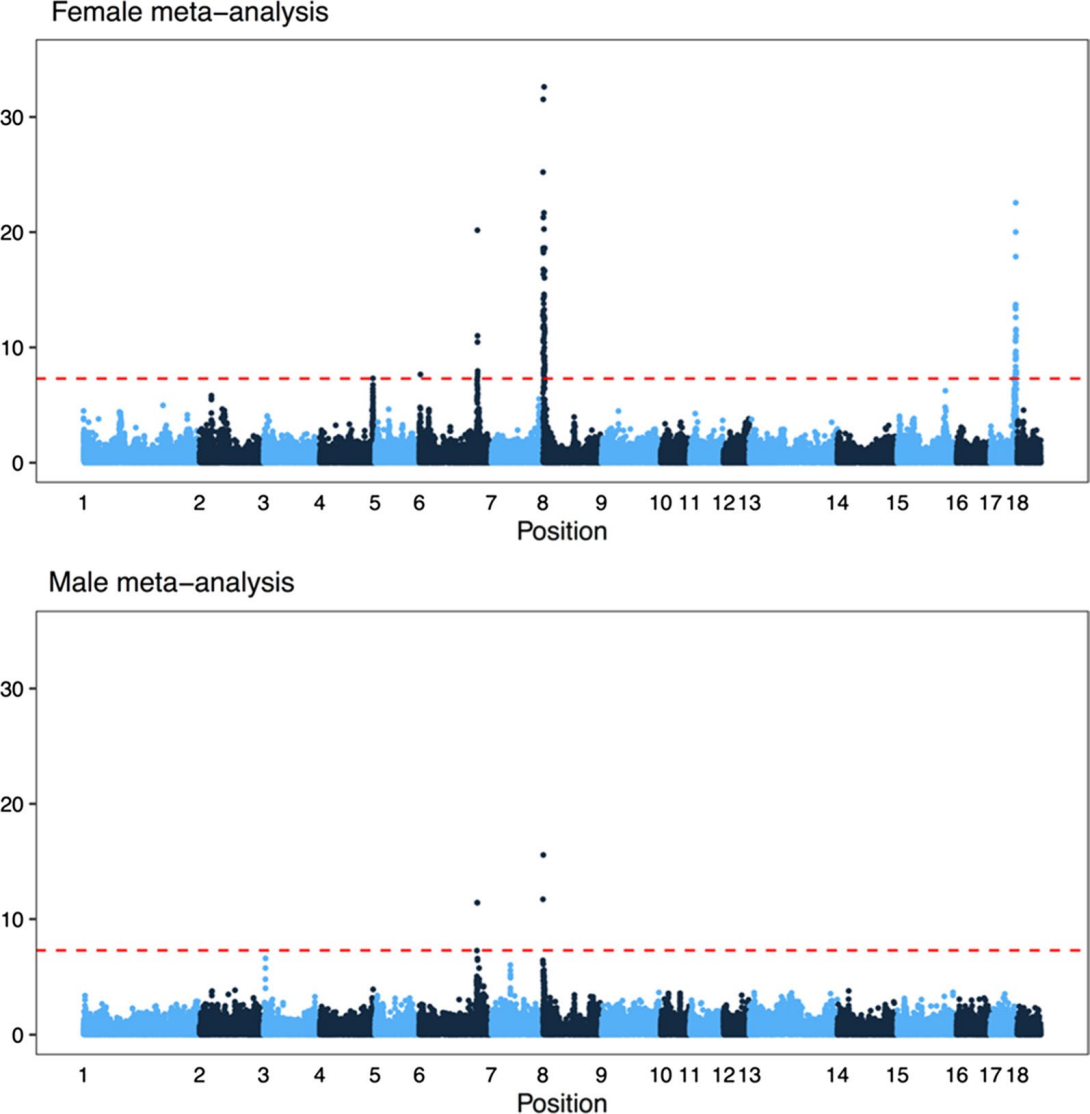
Meta-analysis of genome-wide association studies of genome-wide recombination rates. The subplots are Manhattan plots of the negative logarithm of the *p*-value of association against genomic position, separately for females and males. Alternating colours correspond to chromosomes 1 to 18. Line 7 was excluded from the analysis because of its small number of dams and sires. The dashed red line shows a conventional genome-wide significance threshold of 5 × 10^−8^

**Fig. 8:**
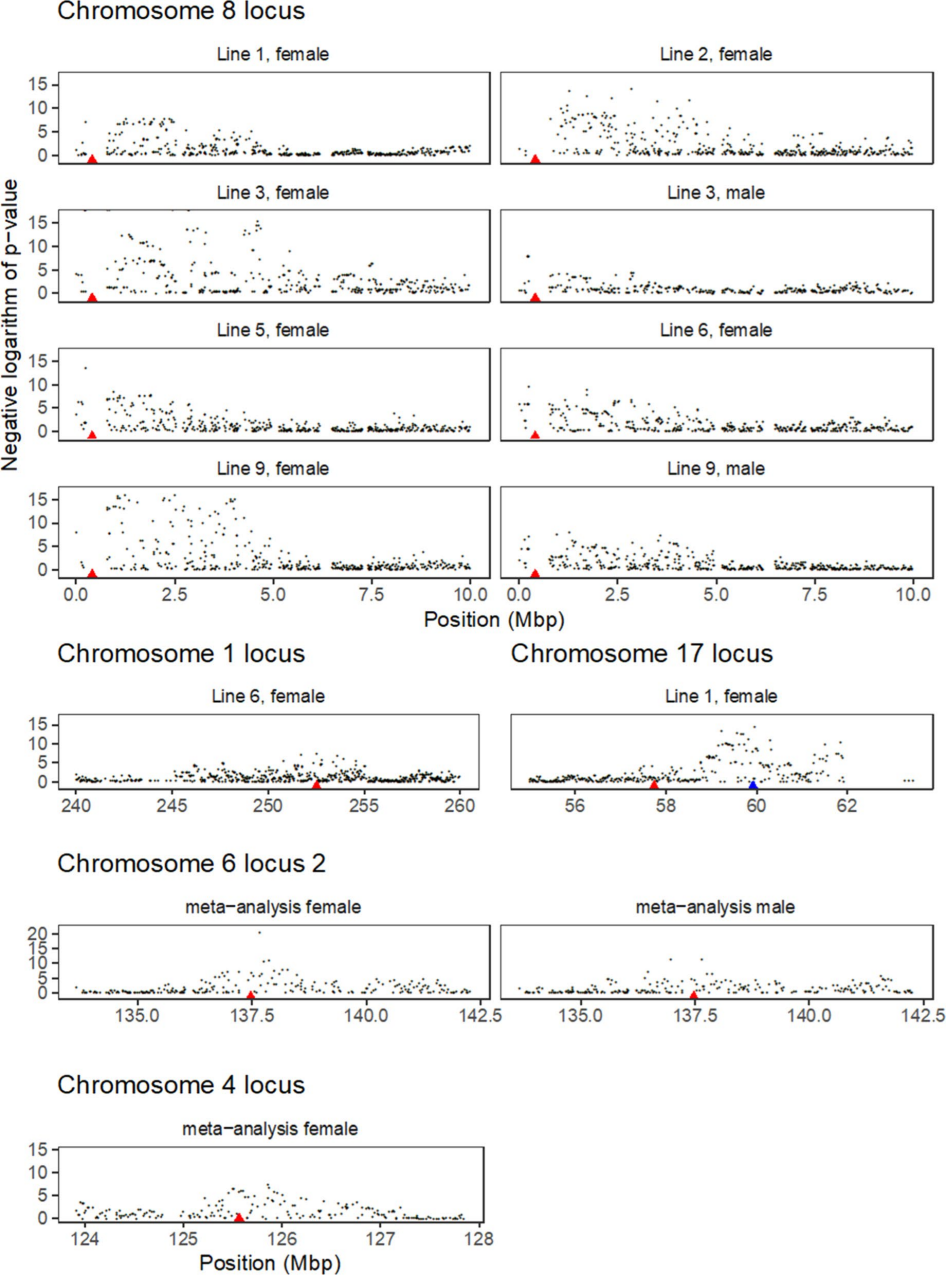
Significant genomic regions for recombination rate that contained candidate genes for recombination. The subplots are Manhattan plots of the negative logarithm of the p-value of association against genomic position, zoomed in to show the region around the significant markers. The red triangles show the locations of *RNF212* (ENSSSCG00000045703) on chromosome 8, *SHOC1* on chromosome 1 (ENSSSCG00000005463), *SPO11* (ENSSSCG00000007502) in red and *SYCP2* in blue on chromosome 17, *MSH4* (ENSSSCG00000003775) on chromosome 6, and *HFM1* (ENSSSCG00000006912) on chromosome 4

The meta-analysis of the genome-wide association studies detected two of the above-mentioned regions (on chromosomes 8 and 17) and three other regions that were not significant in the separate analyses. Figure 7 shows the results of the meta-analysis broken down by sex. Table 4 shows the location of the most significant SNP for each region and the closest gene. In the female meta-analysis, two additional regions were detected on chromosome 6 (with one of these also detected in the male meta-analysis) and one on chromosome 4. Five of the significant regions overlapped with known candidate genes involved in recombination based on [53]. Figure 8 shows details of these significant regions on chromosomes 1, 4, 6, and 17.

**Table 4:** Genome-wide association study hits from the meta-analysis, with position of the lead (most significant) SNP

Chr	Sex	Lead SNP Position	Closest gene to lead SNP
4	Female	125,868,001	ZNF644 (ENSSSCG00000022534)
6	Female	2,754,836	lncRNA (ENSSSCG00000043170)
6	Male	136,977,336	ST6GALNAC3 (ENSSSCG00000024494)
6	Female	137,673,521	SLC44A5 (ENSSSCG00000003777)
8	Male	976,400	NSD2 (ENSSSCG00000008682)
8	Female	2,862,902	SH3TC1 (ENSSSCG00000008722)
17	Female	59,968,884	FAM217B (ENSSSCG00000007531)

*Chr* chromosome number

### Performance of the algorithm with simulated data

**Fig. 9:**
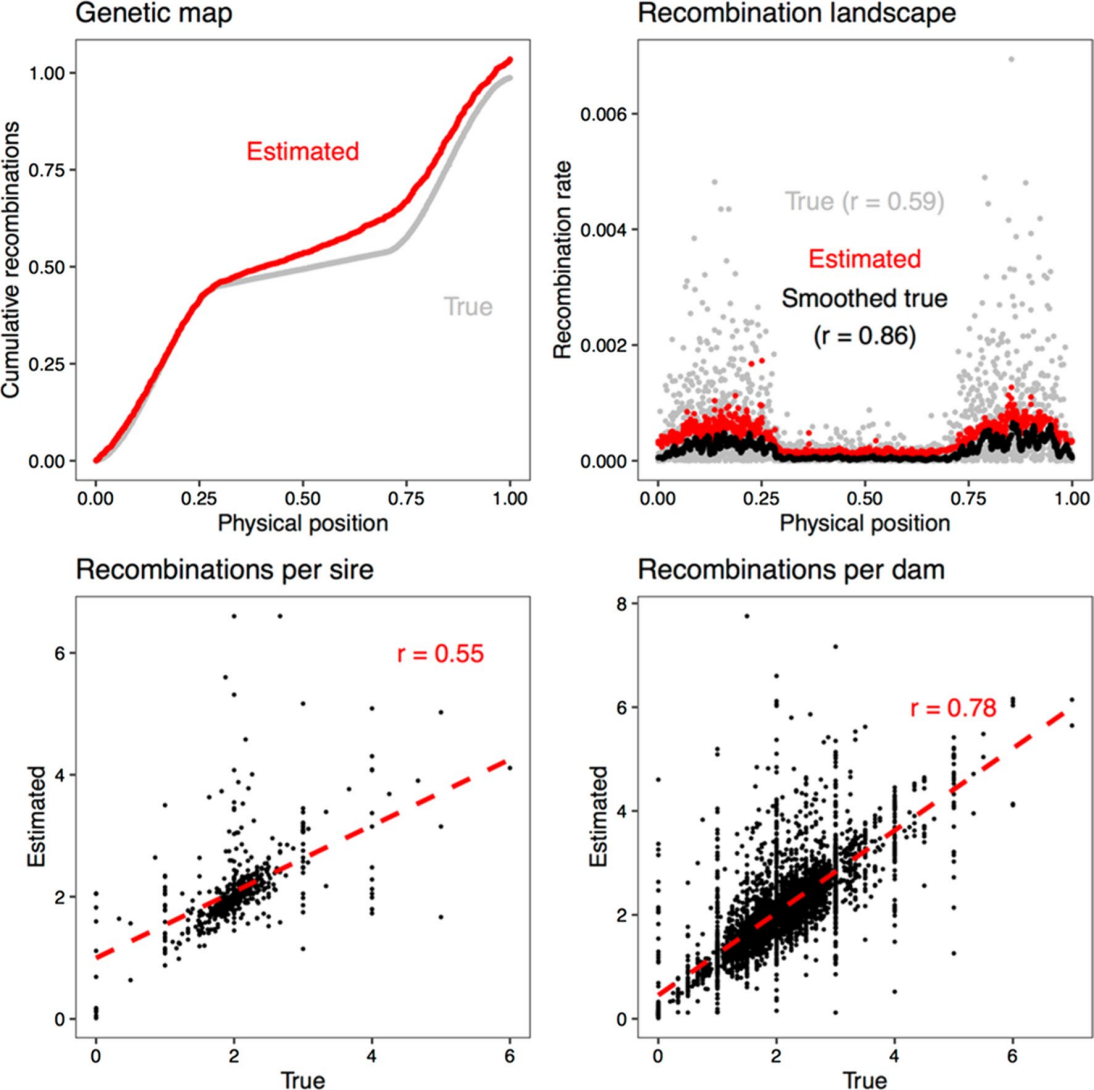
Estimation of recombination rates using simulated data. Cumulative number of recombination events, recombination landscape along the simulated chromosome, and correlation between true and estimated numbers of recombination events in sires and dams. The smoothed values are rolling averages of 50 markers. The red dashed line is the regression line between true and estimated values

We tested the accuracy of the estimated recombination parameters by analysing a simulated dataset. Figure 9 shows the simulated and estimated genetic map length, recombination landscape, and a scatterplot of simulated and estimated numbers of recombination events per individual. Our method slightly overestimated the overall recombination rate when the recombination rate varied along the chromosome. Because of the uncertainty in the location of recombination events, the estimated recombination landscape did not track per-marker recombination rate variation very well (r = 0.59) but captured the smoothed recombination landscape based on 50-SNP windows better (r = 0.86). The accuracy of the estimates of recombination rate at the individual level was higher for dams (r = 0.78) than for sires (r = 0.55).

## Discussion

In this work, we have estimated the variation in recombination rate within the genome and between individuals in nine genotyped commercial pig breeding populations using multilocus iterative peeling. In this section, we discuss three main results: (1) we have confirmed the known features of the pig recombination landscape, but not the previously described correlation with the *PRDM9* consensus motif; (2) we have shown that recombination rate in the pig is genetically variable and associated with alleles at the *RNF212*, *SHOC1*, *SYCP2*, *MSH4*, and *HFM1* genes; and (3) we have demonstrated that multilocus iterative peeling is a compelling method for assessing recombination landscapes from large genotyped pedigrees, but that it tends to overestimate genetic map length.

### Features of the landscape of recombination rate in the pig genome

Our results recover some of the known features of recombination in the pig genome, including the relative chromosome genetic lengths and the marked sexual dimorphism. However, there are two notable exceptions: (1) our estimates of the overall genetic length of chromosomes are greater, and (2) correlations of recombination rates with density of the *PRDM9* consensus binding motif and with density of some repeat classes differed from previously reported estimates.

Regarding exception (1), we obtained total genetic map lengths that ranged from 18.5 to 21.7 M for males and 22.3–25.9 M for females, whereas Tortereau et al. [16] found sex-specific map lengths of 17.8 and 17.5 M for males, and 22.4 and 25.5 M for females (from two different crosses). This difference may be due to overestimation (see below) but also to the higher marker density used and the more complete use of pedigree data, allowing more recombination events to be detected.

Regarding exception (2), we observed that the correlation between recombination rate and density of the *PRDM9* consensus binding motif was lower than previously reported and that the correlation between recombination rate and density of the porcine *PRDM9* motif estimated from the pig PRDM9 amino acid sequence was negative. Because the PRDM9 protein targets recombination events to particular regions, thus determining the locations of a subset of recombination hotspots, the positive correlation between recombination rate and the consensus *PRDM9* motif previously detected by Tortereau et al. [16] is biologically plausible. However, our results are not consistent with this positive correlation, which suggests that we lack the genomic resolution to detect variation at this scale, potentially because we used imputation, in contrast to [16]. Furthermore, ab initio searches of position-specific weight matrices against the genome sequence are known to have a high rate of false positives [57]. Fundamentally, recombination hotspot targeting operates at a much smaller scale than can be estimated using pedigree-based analyses, as used here, which cannot detect hotspots of a few kb (as estimated by population sequencing [6] or by high-density gamete genotyping [58]). Thus, such subtle local variations in recombination rate, like hotspots, could not be detected in our study.

Associations of recombination rate with density of transposable elements varied with the type of transposable elements. We found an overall negative correlation of recombination rate with DNA repeats, in line with estimates reported for other species [4]. The negative correlation of recombination rate with LINEs was stronger than previously reported and the positive correlation of recombination rate with simple DNA repeats was weaker. Another reason for these differences might be that we used the more complete Sscrofa11.1 reference genome [42], which likely better resolves the landscape of repeats in the pig genome than the previous version.

### Genetic variation in autosome-wide recombination rate

Our results on variation in recombination rate in the pig genome agree with the general results in vertebrates, with a low but non-zero heritability and associated genomic regions that overlap with known meiosis-related candidate genes. In particular, these candidate genes are involved in the process that determines whether a double strand break resolves as a crossover or as a non-crossover. The significant region on chromosome 8 is homologous to regions that have been identified to be associated with recombination rate in humans [59–61], cattle [24, 25, 27], sheep [30, 31], and chickens [32], and contains the *RNF212* gene, for which a paralog has also been associated with recombination rate in deer [28, 29]. The RNF212 protein binds to recombination complexes and is essential for crossover formation [62]. The locus on chromosome 1 overlaps with the *SHOC1* gene, which is essential for crossover formation and proper synapsis (i.e. for the physical attachment of homologous chromosomes during meiosis) [63]. While *SHOC1* has not been associated with genetic variation in recombination rate before, it interacts with *TEX11*, which is associated with genome-wide recombination rates in mice [23, 64]. The significant region on chromosome 17 overlaps with the *SYCP2* gene, which is required for assembly of the synaptonemal complex that connects homologous chromosomes [65]. One of the significant regions on chromosome 6 overlaps with the *MSH4* gene, which is essential for proper chromosome pairing during meiosis [66, 67] and has been associated with variation in recombination rate in humans [61] and cattle [24, 27]. Finally, the locus on chromosome 4 overlaps with the *HFM1* gene, which is required for crossover formation [68] and is associated with recombination rate in cattle [24]. The genes *RNF212*, *SHOC1*, *SYCP2,* and *MSH4* are among those recombination-associated genes that have been found to evolve rapidly within mammals [53].

While these genes are suggestive candidates, one should keep in mind that the identified associated regions are large and overlap many genes. As shown in Fig. 8, the significant region on chromosome 8 spans many Mb that contain highly significant SNPs in multiple lines. Within the significant region on chromosome 1, the most significant SNP lies in the *SHOC1* gene. For the significant region on chromosome 6, *MSH4* is about 130 kb away from the most significant SNP in the female meta-analysis and is between the two most significant SNPs in the male meta-analysis. For the significant region on chromosome 4, *HFM1* is about 260 kb away from the most significant SNP in the female meta-analysis. Finally, for the significant region on chromosome 17, the candidate gene *SYCP2* is 13 kb away from the most significant SNP but it also contains the *SPO11* gene, which is located about two Mb away from the most significant SNP. *SPO11* encodes a key enzyme for creating the programmed double-strand breaks that initiate recombination [69] and that is associated with genetic variation in recombination rate in chickens [32].

Our results on recombination rate in the pig genome do not fully agree with those of a recent study by Lozada-Soto et al. [70], who also performed quantitative genetic and genome-wide association studies on recombination rate in the pig. In agreement with our results, they found that recombination rate has a low heritability, and that average recombination rate differed between populations. Their genome-wide association study identified several regions, but none of these overlapped with those identified in our study, nor did they include any previously known candidate genes for recombination rate [70]. These discrepancies may be due to methodological differences, the limited power of genome-wide association analyses of recombination rate, or to genuine genetic differences.

We observed differences in recombination rate between lines, which may be due to genetic differences. Given that livestock populations have relatively small effective population sizes, and assuming that variation in recombination rate has a rather simple genetic architecture, differences in recombination rate between lines might very well be due to genetic differences that have become fixed by chance. At the same time, all the lines studied here showed evidence of comparable genetic variation in recombination rate, and there was evidence that the major quantitative trait locus for recombination rate on chromosome 8 segregates in most lines.

One limitation of our study, and a possible avenue for future research, is that the study does not include the X chromosome; to do this a further development of our recombination inference method would be required. Both the association study and the estimation of recombination rates pertain only to the autosomes. The pig X chromosome is known to display regional variation in recombination rate, including one long “cold spot” of very low recombination rate [71], and differences in recombination rate between families and breeds [72]. A recent study of recombination rates on the X chromosome in cattle [73] suggested that autosomal and X chromosomal recombination rates were highly correlated in females, but that the male-specific X chromosomal recombination rate might be a distinct trait, since it was lowly correlated with male autosomal recombination rate, although it was heritable. Such a sex-difference in genetic architecture is biologically plausible, as male recombination on the X chromosome can only occur in the pseudo-autosomal regions. Furthermore, the X chromosome houses one of the most compelling candidate genes for recombination rate, i.e. *TEX11*, a meiosis gene that evolves rapidly in vertebrates [53] and is associated with azoospermia and failure of meiotic synapsis in humans and mice [22, 64].

A higher recombination rate could be beneficial for breeding, because it would reduce linkage disequilibrium between causative variants and release genetic variance. Simulations have suggested that a substantial increase in the genome-wide recombination rate could increase genetic gain [74]. Based on our results, we were able to approximate how much breeding could increase recombination rate. First, we used the breeders’ equation [75] to predict response to selection $R$, treating genome-wide recombination as a quantitative trait. $R$ is equal to the heritability multiplied by the selection differential $S$, which is the difference between the population mean $\mu$ and the mean of the selected individuals ${\mu }_{selected}$: $R={h}^{2} S= {h}^{2} ({\mu }_{selected}- \mu )$

Using the distribution of estimated genome-wide recombination rates for the males in the largest line, the mean was 0.904 cM/Mb. If we selected the 10, 20 or 30% individuals with the highest recombination rate, the mean of the selected individuals ${\mu }_{selected}$ would be 1.22 cM/Mb, 1.15 cM/Mb, and 1.11 cM/Mb, respectively. Assuming a heritability of 0.05, comparable to our estimates, this would result in selection responses of 0.016 cM/Mb, 0.012 cM/Mb and 0.010 cM/Mb, respectively. Thus, relative to the average recombination rate, this would result in increases of 1.7, 1.3 and 1.1%, respectively, for one round of selection.

Second, we estimated the increase in recombination rate if the favourable allele for the major quantitative trait locus on chromosome 8 that we detected in most of the lines was fixed. Again, using estimates from the largest line, the estimate of the additive effect of the locus was 0.0271 cM/Mb (averaging the male and female estimates) and the frequency of the favourable allele was 0.332 (weighted average of males and females). Thus, fixing this locus would increase the recombination rate b﻿y﻿ $0.0271\times\left(1-0.332\right)=0.018 \mathrm{cM}/\mathrm{Mb}$, an increase of the genome-wide recombination rate by about 2%.

Compared to the simulation results of [74], which suggested that a doubling or more of the genome-wide recombination rate results in substantial genetic gains, our results suggest that breeding for higher genome-wide recombination rate is not a practical alternative to improve genetic gain. There may be other potential avenues, such as introducing targeted recombination events in favourable locations through biotechnology [76, 77].

### Inference of recombination rate by multilocus peeling

In this study, we have used multilocus iterative peeling to estimate recombination rates. Inferring recombination events and imputing genotypes simultaneously allows the use of large datasets without requiring high-density genotyping. However, the genotyping density does put a limitation on the resolution at which recombination events can be localised on the genome. In our simulation study, we found that multilocus iterative peeling could estimate the number of recombination events per individual with an accuracy of 0.7 for dams and 0.5 for sires, and the average recombination landscape along a chromosome. This is consistent with our analysis of the pig genome, for which we confirm previously known features of its average recombination landscape. However, the simulation results also show that our method overestimates the total genetic map length, which is also evident from comparisons with previously published estimates [16].

Multilocus iterative peeling is a compelling technique for estimating recombination rate in large pedigree populations: it scales well to massive livestock pedigrees (i.e. more than 150,000 individuals), does not require pre-phasing of the data, and handles individuals that may be genotyped on a range of platforms without requiring non-overlapping variants to be imputed beforehand. We evaluated the accuracy of imputation with multilocus iterative peeling with simulated data based on four of the pedigrees included in this study and found that it was high for individuals that were genotyped with at least 10,000 SNPs [40].

One of the major downsides of multilocus iterative peeling is that it requires multiple generations of genotyped individuals to accurately phase and impute genotypes, and to estimate the recombination rate. Although this information may be available in pig or chicken breeding programmes [38, 78], and for some wild populations [30], this may not be always the case. In addition, the observed overestimation of the length of the genetic map suggests that estimates may not be accurate. However, the multilocus iterative peeling method is able to recover broad patterns of recombination events within chromosomes and between individuals.

## Conclusions

By analysing 150,000 individuals from nine pig pedigrees, we were able to recover broad-scale patterns in genetic map lengths, landscapes of recombination rates, and sex differences in recombination rates. We also found that recombination rate had a low, but non-zero heritability and, by performing a genome-wide association study, we detected six regions that are associated with recombination rate. Our results highlight that large-scale pedigree and genomic data, as routinely collected in many closely-managed populations, can be used to infer and understand recombination and variation in recombination rate along the genome.

## Supplementary Information

**Additional file 1: Figure S1.** Predicted porcine PRDM9 binding site from the amino acid sequence, with reverse complement and the canonical PRDM9 motif for comparison. The sequence logos were generated with the online predictor of [45].

**Additional file 2: Table S1.** Male map of the landscape of pig recombination rate in 1-Mb windows

**Additional file 3: Table S2.** Female map of the landscape of pig recombination rate in 1-Mb windows.

**Additional file 4: Table S3.** Sex-averaged map of the landscape of pig recombination rate in 1-Mb windows.

## Data Availability

The datasets generated and analysed in this study are derived from the PIC breeding programme, and are not publicly available.
