# Supplementary figures and images for: Genetic variation in recombination rate in the pig
Source: Genet Sel Evol. 2021 Jun 25;53:54. doi: 10.1186/s12711-021-00643-0 (PMC8235837; doi:10.1186/s12711-021-00643-0)

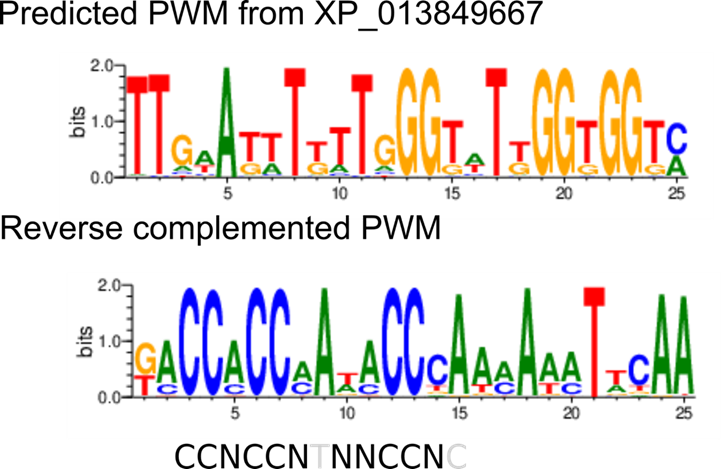

Supplement: Supplementary file 1 — Additional file 1: Figure S1. Predicted porcine PRDM9 binding site from the amino acid sequence, with reverse complement and the canonical PRDM9 motif for comparison. The sequence logos were generated with the online predictor of [45]. [file 12711_2021_643_MOESM1_ESM.docx]
